# Occlusion of Inferior Vena Cava: A Singular Presentation of Abdominal Aortic Aneurysm

**DOI:** 10.1155/2009/827954

**Published:** 2009-12-21

**Authors:** Daniel Brandão, João Correia Simões, Alexandra Canedo, Miguel Maia, Joana Ferreira, Sandrina Braga, João Vasconcelos, Guedes Vaz

**Affiliations:** ^1^Angiology and Vascular Surgery Department, Vila Nova de Gaia/Espinho Hospital Center, 4434-502 Vila Nova de Gaia, Portugal; ^2^Angiology and Vascular Surgery Department, Alto Ave Hospital Center, 4835-044 Guimarães, Portugal

## Abstract

Even though the majority of abdominal aortic aneurysm s(AAAs) are asymptomatic, they can occasionally manifest as a result of adjacent structures involvement. Although the most frequent venous complication of AAA is rupture into the inferior vena cava (IVC), venous compression can infrequently occur. The authors report a particularly rare case of compression and thrombosis of the IVC by AAA. Patient was treated by preoperative placement of an IVC filter to impede pulmonary embolism and subsequently by open surgical repair. Besides discussing the circumstances associated with IVC compression by AAA, the authors also discuss the treatment strategy adopted and the possible alternatives.

## 1. Manuscript

 Even though the vast majority of abdominal aortic aneurysms (AAAs) are asymptomatic, they may indirectly present with symptoms related to compression of adjacent structures, such as gut, ureter, or major veins [1]. In this context, the compression of Inferior Vena Cava (IVC) by an AAA is an extremely rare situation, needing a high level of suspicion to be diagnosed. Accordingly, we present a case of IVC collapse and caudal thrombosis as a result of extrinsic compression by an AAA. 

## 2. Case Report

**Figure 1:**
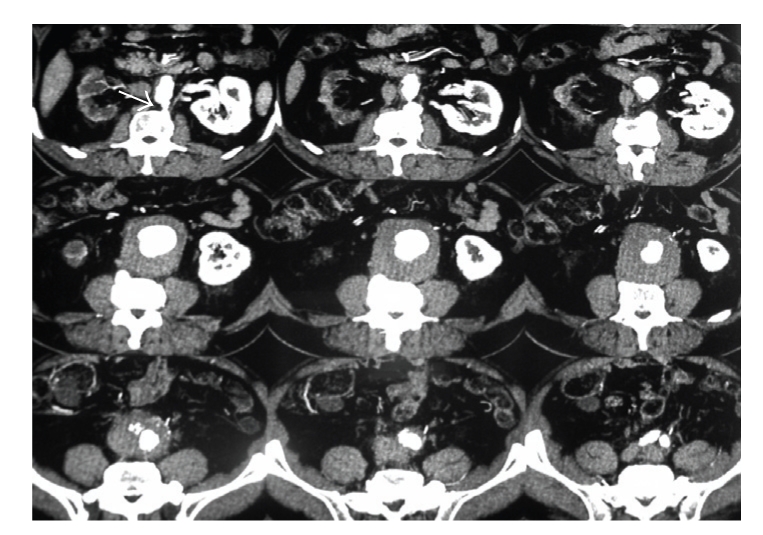
Cross-sectional CT scan demonstrates large abdominal aortic aneurysm compressing Inferior Vena Cava (IVC). The arrow points to juxta-renal IVC, the arrow head points to the virtually occluded IVC, and the line to the thrombosed common iliac veins.

**Figure 2:**
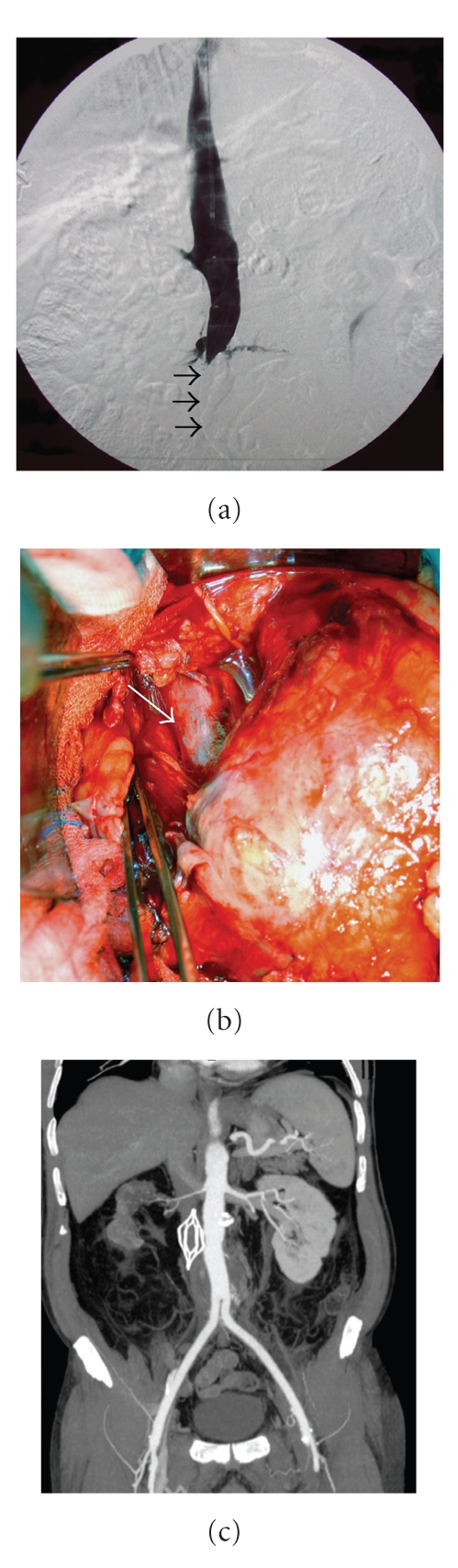
(a) Inferior vena cavagram performed through jugular approach demonstrates collapse of IVC at the AAA level (Black arrows). (b) Operative photograph shows IVC (white arrow) compressed medially and anteriorly by AAA. (c) Eight-month-postoperative coronal CT reconstruction shows patency of the vascular graft and of the IVC at filter level and cranially.

A 64-year-old man was referred to our department with eight-day progressive edema and pain of the inferior limbs, with predominance on the right side. He reported no history of prior venous thromboembolic event, recent trauma, surgery, malignancy, or travel. He had a medical history significant for type 2 diabetes mellitus, dyslipidemia, smoking, and a prior hospitalization for a stroke, from which he had a complete recovery. Besides confirming the presence of a significant pitting edema reaching the proximal thigh bilaterally, even though more prominent on the right side, physical examination revealed a pulsatile abdominal mass. An ultrasound was immediately performed. It showed the presence of extensive bilateral lower extremity deep vein thrombosis in the common iliac, external iliac, femoral, and popliteal venous system. It also confirmed the presence of 8.7 cm diameter AAA. He was admitted for anticoagulant therapy and further evaluation of the AAA. Computed Tomography (CT) demonstrated a compression of the IVC by an AAA moderately developing to the right, with apparent collapse and caudal thrombosis and a nonfunctioning right kidney (urea and creatinine blood levels remained within a normal range) (Figure 1). Subsequently, an IVC filter was placed infrarenally through transjugular access. Inferior vena cavagram performed before the deployment of the filter, confirmed the collapse of the IVC (Figure 2(a)). Anticoagulation was suspended immediately before AAA surgical repair. After a transperitoneal approach, the aneurismal sac was exposed in the retroperitoneum. A meticulous dissection of the aneurysm was required until reaching the neck at the left renal vein level because of the proximity of IVC and the presence of abnormally dilated retroperitoneal veins. No macroscopic signs of inflammatory AAA were observed (Figure 2(b)). After having opened the aneurysm sac, the thrombus was removed in an attempt to decompress the IVC. The common femoral arteries were then dissected bilaterally and a 14/7 mm knitted Dacron aortofemoral graft was placed after heparinization. The proximal anastomosis was constructed end-to-end whereas distal anastomoses were both carried out end-to-side to the common femoral arteries. 

Anticoagulation was resumed postoperatively and maintained for 6 months. The patient was discharged after an uneventful recovery and followedup in the outpatient clinic. The lower extremity edema progressively diminished. An abdominal CT scan, obtained 8 months postoperatively, demonstrated patency of the vascular graft and of the IVC at filter level and cranially (Figure 2(c)). Iliac veins were partially recanalized, but IVC adjacent to the remaining aneurysm sac continued apparently collapsed. 

## 3. Discussion

Involvement of adjacent structures can seldom be the presentation of an AAA, particularly inflammatory AAA. Although the most widely recognized venous complication of AAA is rupture into the IVC, AAA can also manifest by compression of neighbouring veins [2]. A review of the English literature demonstrated nine reported cases of IVC compression by an AAA so far. The majority (five) has been described in a context of inflammatory AAA, which are usually much more prone to adhere to adjacent structures [2–7]. Meanwhile, typical characteristics of inflammatory AAA like marked thickening of the aneurysm wall, fibrosis, and desmoplasia of the adjacent retroperitoneum and rigid adherence of local structures to the anterior aneurysm wall were not evident in the present case [8]. IVC compression is generally associated with large dimension aneurysms; as a result, it can represent an indicator of increased AAA rupture risk. IVC involvement also seems to be more frequently associated with AAA developing to the right [2]. 

Considering the high rupture risk of an 8.7 cm AAA and the fact that anticoagulation could potentially increase the morbidity and mortality associated with a possible rupture, a surgical treatment of AAA was carried out. The risk of pulmonary embolism during the intervention due to anticoagulation interruption, local manipulation, and hemodynamic variability must be limited. As a result, an IVC filter was preoperatively placed in an infrarenal position as the clots did not approach renal veins, which was an additional concern in a patient with a single functioning kidney. Intraoperative alternatives, like IVC clip application, would lead to an extended dissection which could be associated with a potentially increased blood loss because of the enlarged and prone to rupture retroperitoneal veins. Endovascular repair of the AAA was not considered because it would probably not allow decompression of the IVC. Meanwhile, the persistent collapse of the IVC adjacent to the remaining AAA sac may be a result of already established adhesions between IVC walls. 

In conclusion, despite anatomic proximity of aorta and IVC, AAA is a very rare cause of symptomatic IVC compression and thrombosis. In surgery of an AAA associated with IVC thrombosis, it is crucial to avoid a pulmonary embolism and consequently an IVC filter should be placed preoperatively. In those situations, dissection is quite challenging due to enlarged collateral veins present in retroperitoneum and should be as limited as possible.
